# ASFV pS183L protein negatively regulates RLR-mediated antiviral signalling by blocking MDA5 oligomerisation

**DOI:** 10.1186/s13567-025-01488-x

**Published:** 2025-03-31

**Authors:** Huan Chen, Qun Yu, Xiaoyu Gao, Tao Huang, Chenyi Bao, Jiaona Guo, Zhenzhong Wang, Jiaxuan Lv, Jianjun Dai, Lorne A. Babiuk, Xingqi Zou, Yong-Sam Jung, Yingjuan Qian

**Affiliations:** 1 Laboratory of Emerging Infectious Diseases and One Health, College of Veterinary Medicine, Nanjing Agricultural University, Nanjing, Jiangsu Province, China; 2 MOE Joint International Research Laboratory of Animal Health and Food Safety, College of Veterinary Medicine, Nanjing Agricultural University, Nanjing, Jiangsu Province China; 3 School of Life Science and Technology, China Pharmaceutical University, Nanjing, China; 4 University of Alberta, Edmonton, AB Canada; 5 China/WOAH Reference Laboratory for Classical Swine Fever, China Institute of Veterinary Drug Control, Beijing, China; 6 Veterinary Bio-Pharmaceutical, Jiangsu Key Laboratory for High-Tech Research and Development of Veterinary Biopharmaceuticals, Jiangsu Agri-Animal Husbandry Vocational College, Taizhou, Jiangsu China

**Keywords:** African swine fever virus, pS183L, MDA5, oligomerisation, IFN-β

## Abstract

The retinoic acid-inducible gene I (RIG-I)-like receptors (RLRs) are major sensors against viral infection, but their roles in DNA virus infection largely remain unknown. This study found that a previously uncharacterised protein, pS183L, negatively regulates RLR signalling by suppressing MDA5 oligomerisation. Specifically, we showed that the overexpression of pS183L suppresses MDA5 but not cGAS-STING or RIG-I-induced IFN-β activation. Consistently, pS183L inhibited high molecular weight poly (I:C) activated IFN-β production. Furthermore, we demonstrated that pS183L interacts with CARDs and the MDA5 Helicase domain, consequently blocking MDA5 oligomerisation and the MDA5-MAVS interaction. Taken together, we concluded that pS183L blocks MDA5 oligomerisation through protein–protein interaction and thus disrupts MDA5-mediated IFN-β signalling.

**Supplementary Information:**

The online version contains supplementary material available at 10.1186/s13567-025-01488-x.

## Introduction

African swine fever (ASF), caused by the African swine fever virus (ASFV), is an acute haemorrhagic disease affecting domestic pigs and wild boars. Since its identification in Kenya in 1921, ASF has spread across Africa, Europe, and Asia [1]. The highly lethal Genotype II ASFV strain was first reported in Shenyang, China, in August 2018. Since then, it has spread to almost all Chinese provinces, leading to significant economic losses [2, 3]. Subsequent surveillance studies showed that lower-virulent genotype II strains emerged in China due to natural mutations in the genomes of high-virulence ASFV strains [4]. In 2021, genotype I strains similar to NH/P68 and OURT88/3 were identified in China. These strains exhibited lower virulence, longer latent periods, and stronger transmissibility than genotype II strains, posing new disease prevention and control challenges [5]. Even more concerning, highly lethal genotype I and II ASFV recombinants were discovered in China in 2021 and 2022 [6].

ASFV, the only member of the *Asfaviridae* family, is a double-stranded DNA virus. Its genome ranges from 170 to 190 kbp and encodes between 150 and 200 viral proteins, including more than 50 structural and 100 non-structural proteins [7, 8]. The proteins encoded by the ASFV genome are involved in replication, virion assembly, icosahedral formation, immune evasion, etc. For example, DNA polymerase type B (pG1211R), DNA topoisomerase type II (pP1192R), DNA ligase (pNP419L), DNA primase (pC962R), and histone-like DNA-binding protein (pA104R) have been shown to participate in ASFV replication independently of host cells [9]. The non-structural protein B602L and transmembrane protein p17 are reported to participate in virion assembly or icosahedral formation [10, 11].

Furthermore, different ASFV proteins utilise diverse immune escape strategies. For example, pMGF505-7R, pMGF505-11R, pH240R, and pE184L are reported to inhibit the cGAS-STING mediated IFN-β production by targeting STING [12–15]. Research indicates that pI267L impairs the innate antiviral responses activated by RNA polymerase III-RIG-I (retinoic acid-inducible gene I) [16]. Additionally, pMGF505-7R and pH240 inhibit interleukin-1β (IL-1β) production upon targeting the NLRP3 inflammasome pathway [17, 18]. Nonetheless, nearly half of the viral protein functions remain unknown, including immune regulation proteins, which challenge the development of effective vaccines and antiviral drugs.

The type I interferon (IFN-I) response is the host’s first primary defence mechanism upon a viral infection. It is triggered by pattern recognition receptors (PRRs) that recognise pathogen-associated molecular patterns (PAMPs). PRRs are classes of receptors that include RIG-I-like receptors (RLRs), nucleotide-binding and oligomerisation domain (NOD-like) receptors (NLRs), toll-like receptors (TLRs), and Cyclic GMP-AMP synthase (cGAS).

Recently, the cytoplasmic DNA sensor cGAS has been exclusively studied during viral infections, particularly in DNA viruses such as HSV-1, KSHV-1, ASFV, and PRV [19–23]. Interestingly, it was reported that RLR signalling is also activated by DNA virus infection. For example, herpes simplex virus 1 (HSV-1) infection is mainly recognised by MDA5 (melanoma differentiation-associated protein 5) to activate the IFN-β production [24, 25]. It has also been reported that the Epstein-Barr virus (EBV)-encoded small RNAs are recognised by RIG-I and activate type I IFN signalling in EBV-infected cells [26]. Furthermore, a recent report revealed that the RIG-I-mediated immune response is activated by host-derived RNAs upon HSV-1 infection [25]. Similarly, RLRs are activated by host-derived RNAs to restrict Kaposi sarcoma-associated herpes virus (KSHV) lytic reactivation [27]. Another study has established that RIG-I and MDA5 mRNA levels are up-regulated upon ASFV infection [28]. ASFV glycoprotein I329L has also been shown to inhibit the dsRNA-stimulated activation of NF-κB and IRF3 (interferon regulatory factor 3) [29]. Nevertheless, the mechanism by which ASFV infection activates RLR signalling and the specific ASFV proteins that regulate the RLR pathway are still largely unknown.

RIG-I and MDA5 belong to the RLR family of cytoplasmic viral receptors for RNA and have similar structures. They also have the same signalling adaptor, mitochondrial antiviral-signalling protein (MAVS). RIG-I recognises short dsRNA with 5’-ppp caps, whereas MDA5 recognises the internal duplex structure of longer double-stranded RNA (dsRNA) [30]. When binding to the dsRNA, RIG-I/MDA5 conformational rearrangement occurs, inducing the formation of RIG-I or MDA5 oligomers. Subsequently, RIG-I or MDA5 oligomers translocate to the mitochondrion and recruit MAVS, which stimulates the phosphorylation of TANK binding kinase 1 (TBK1) and IkappaB kinase (IKK). The phosphorylated TBK1 and IKK then activate IRF3 or NF-κB nucleus translocation to initiate interferon transcription.

Our study found that pS183L negatively affects RLR-mediated antiviral signalling by inhibiting MDA5 oligomerisation through protein–protein interactions. Importantly, pS183L is a previously uncharacterised protein of ASFV. Exploring the role of uncharacterised ASFV proteins involved in regulating host IFN response will enrich the knowledge of virus infection and provide new targets for vaccine development.

## Materials and methods

### Cells, antibodies, and reagents

PK15 and 293 T cells were cultured in Dulbecco’s modified Eagle’s medium (DMEM, Gibco-BRL), supplemented with 8% foetal bovine serum (PAN-Biotech, Dorset, GB) and 1% penicillin–streptomycin (Beyotime Biotechnology, Shanghai, China) at 37 ℃ in a 5% CO_2_ incubator.

The mouse anti-Flag antibody was purchased from Sigma-Aldrich (St. Louis, MO, USA). Rabbit anti-phospho-TBK1, rabbit anti-phospho-IκBα and rabbit anti-IκBα antibodies were purchased from Cell Signaling Technology (Beverly, USA). Rabbit anti-HA (haemagglutinin), mouse anti-TBK1, rabbit anti-IRF3, and mouse anti-actin antibodies were purchased from Proteintech (Wuhan, China). The mouse anti-Myc antibody was purchased from GeneTex (Irvine, CA, USA), while the rabbit anti-phospho-IRF3 antibody was purchased from Abcam (Cambridge, MA, USA).

A polyclonal antibody against pS183L was generated in mice by immunisation with purified recombinant pS183L (10–168 aa) truncate protein. HRP-conjugated goat anti-mouse IgG(H + L) antibody was obtained from Millipore (Billerica, MA, USA). The Alexa Fluor 488-conjugated goat anti-rabbit IgG and Alexa Fluor 555-conjugated goat anti-mouse IgG were purchased from Thermo Fisher Scientific (MA, USA). Lastly, the high molecular weight (HMW) poly(I:C) and low molecular weight (LMW) poly(I:C) were obtained from InvivoGen (San Diego, CA, USA).

### Plasmids

S183L was a mammalian codon optimised and synthesised by Tsingke Biotechnology, then cloned into pcDNA3-2XFlag, pcDNA3-Myc, and pcDNA4-HA. RIG-I (NM_213804.2), MDA5 (NM_001100194.1), and MAVS (NM_001097429.1) were amplified from the cDNA of PK15 and cloned into pcDNA4-HA or pCAGGS-HA. pcDNA4-HA-cGAS, pcDNA3-2XFlag-STING, and pcDNA4-HA-TBK1 were generated as described previously [31]. The luciferase reporter plasmids employed in this study were as described in a previous study [32]. The truncates of MDA5, CARDs (1-202aa), helicase (203-886aa), and C-terminal domain (CTD) (887-1023aa) were amplified from pCAGGS-HA-MDA5 and subsequently cloned into pcDNA3-2XFlag.

### Dual-luciferase reporter assay

Before transfection, 293 T or PK15 cells were seeded on 24-well plates overnight at a density of 1 × 10^5^ cells/well. The cells were then co-transfected with pGL3-Basic-IFN-β-Luc, pCMV-RL, pcDNA3-2XFlag-S183L, and RIG-I/MDA5/MAVS/TBK1 by Lipofectamine 2000 reagent (Thermo Fisher Scientific) for 24 h. Luciferase activities were measured using a dual-luciferase assay kit (Promega, Madison, WI, USA) and an MD SpectraMax iD5 instrument.

### RNA isolation and RT-qPCR

Total RNA was extracted from the PK15 cells with a Simply P Total RNA Extraction kit (Bioer Technology, China). Using the HiScript II Q RT kit (Vazyme Biotech, China), 100 ng RNA was used for reverse transcription. The PK15 cells were then seeded on 12-well plates overnight at a density of 1 × 10^5^ cells/well before transfection. The cells were subsequently transfected with pcDNA3-2XFlag-S183L (2 μg) for 24 h. This process was followed by high molecular weight poly(I:C) (10 μg/mL) or low molecular weight poly(I:C) (10 μg/mL) transfection for 1 or 4 h.

The primers for the polymerase chain reaction (PCR) of the immune-related genes, MDA5 and RIG-I, are listed in Table 1. Quantitative real-time PCR was performed using SYBR green-based (Vazyme Biotech, China), and the expression levels of the immune-related genes were normalised to the level of actin using the Comparative CT Method (ΔΔCT Method). All samples were performed thrice.

**Table 1:** **The primers used for RT-qPCR or semiquantitative PCR**

Name	Sequence
sus-ISG54 forward	5′- GCACAGCAATCATGAGTGAGAC-3′
sus- ISG54 reverse	5′- CTGGCCCCTGCAGTCTTTTA -3′
sus-TNFα forward	5′- CCACCAACGTTTTCCTCACT-3′
sus-TNFα reverse	5′- CCCAGGTAGATGGGTTCGTA-3′
sus-IFN-β forward	5′- CTGGCTGGAATGAAACCGTC-3′
sus-IFN-β reverse	5′- AATGGTCATGTCTCCCCTGG-3′
sus-MDA5 forward	5′- CCAGAAGTTGTCAAGTCTTGTG-3′
sus-MDA5 reverse	5′- CCTGGTGAGGCTGTTAGTCC-3′
sus-RIG-I forward	5′- GGTTGGAGATGCTTTCAGGGA-3′
sus-RIG-I reverse	5′- GCAGTCTGGCCTAGCACAATA-3′
sus-Actin forward	5′- GAGACCTTCAACACCCCAGCCATG-3′
sus-Actin reverse	5′- GCGACGTAGCACAGCTTCTCCTTG-3′

### Immunofluorescence assay

The 293 T cells were grown on coverslips and transfected with HA-tagged MDA5 and Flag-tagged with S183L or empty vector. The cells were then fixed with 4% formaldehyde and 0.1% Triton X-100 at room temperature for 30 min, 24 h after transfection. After washing with glycine-phosphate buffered saline (PBS), the cells were blocked with 3% bovine serum albumin (BSA) in PBS for 1 h at room temperature. Next, the cells were incubated with anti-Flag (1:200) and anti-HA (1:200) antibodies at 37 ℃ for 1 h. After washing with PBS thrice, the cells were incubated with the indicated secondary antibodies (1:500) at 37 ℃ for 30 min. The nuclei were stained with 4,6-diamidino-2-phenylindole (DAPI), and images were captured using a Nikon fluorescence microscope (TS100-F; DSRi2).

### Co-immunoprecipitation and western blotting

Before being transfected with the described plasmids for 24 h, 293 T cells were cultured in 3.5 cm dishes. The cells were then lysed with lysis buffer (50 mM Tris PH 7.5, 150 mM NaCl, and 1% NP-40 supplemented with protease inhibitor). Following 10 min of centrifuging at 4 ℃, the lysates were co-incubated with anti-Flag antibodies or IgG control and protein A/G magnetic beads overnight at 4 ℃. After being washed eight times with ice-cold lysis buffer, proteins were eluted with a 2XSDS sample buffer and analysed by western blotting. The samples were separated using 8–12% sodium dodecyl sulfate (SDS)-PAGE for western blotting and transferred to a nitrocellulose membrane (NCM). The NCM was blocked in 3% skim milk for 30 min at room temperature, followed by incubation with primary antibodies at 4 ℃ overnight and then with corresponding secondary antibodies at 4 ℃ for 4 h.

### Semi-denaturing detergent agarose gel electrophoresis (SDD-AGE)

The cells transfected with the indicated plasmids were collected in lysis buffer (0.5% Triton X-100, 50 mM Tris pH 7.5, 150 mM NaCl, 10% glycerol) [33] and subsequently eluted with 5X SDD-AGE loading buffer (2.5X TBE, 2.5% SDS, 25% glycerol, 0.0025% Bromophenol blue) at 42 ℃ for 10 min.

Samples were separated in 1.5% agarose gel in 0.1% SDS in Tris–borate-EDTA (TBE) buffer (0.5X TBE, 0.1% SDS) at 80 V for 120 min on ice. The proteins were then transferred to the NCM and incubated with anti-HA and secondary antibodies. The whole-cell lysates were collected and analysed using western blotting.

### ASFV infection

The ASFV HuB/HH/2019 strain [34] was propagated in alveolar macrophages (PAMs). Briefly, 3 × 10^6^ PAM cells were seeded into 3.5 cm dishes and incubated for 24 h. The cells were infected with ASFV (MOI, 1) in serum-free RPMI 1640 medium at 37 °C for 2 h, then washed with PBS and maintained in 10% foetal bovine serum (FBS) RPMI 1640 medium. After 24 h of infection, the cells were collected for co-immunoprecipitation assays.

### Statistical analysis

All the experiments were performed thrice. Statistical analyses were performed using Student’s *t*-test in GraphPad 9.0 software. A *Ρ* value of less than 0.05 was considered statistically significant. *P < 0.05, **P < 0.01, ***P < 0.001.

## Results

### ASFV pS183L attenuates MDA5-mediated IFN-β activation

As a large double-stranded DNA virus, it is well known that ASFV infection activates the cGAS-STING signalling to induce the type I interferon response [21]. Furthermore, reports indicate that the transcription of RIG-I and MDA5 is activated during the early stages of ASFV infection but is suppressed in the later stages [28, 35]. Recently, RNAs extracted from PK15 cells transfected with the ASFV AT-rich genome (ASFV-AT) have been reported to activate the IFN-β promoter. Interestingly, IFN-β production stimulated by ASFV-AT can be down-regulated by a specific inhibitor of RNA Pol-III (ML-60218), suggesting that the ASFV-AT-rich genome activates RNA Pol-III-RIG-I-mediated innate immunity [16]. However, ASFV proteins that regulate the MDA5-mediated pathway remain unknown.

**Figure 1:**
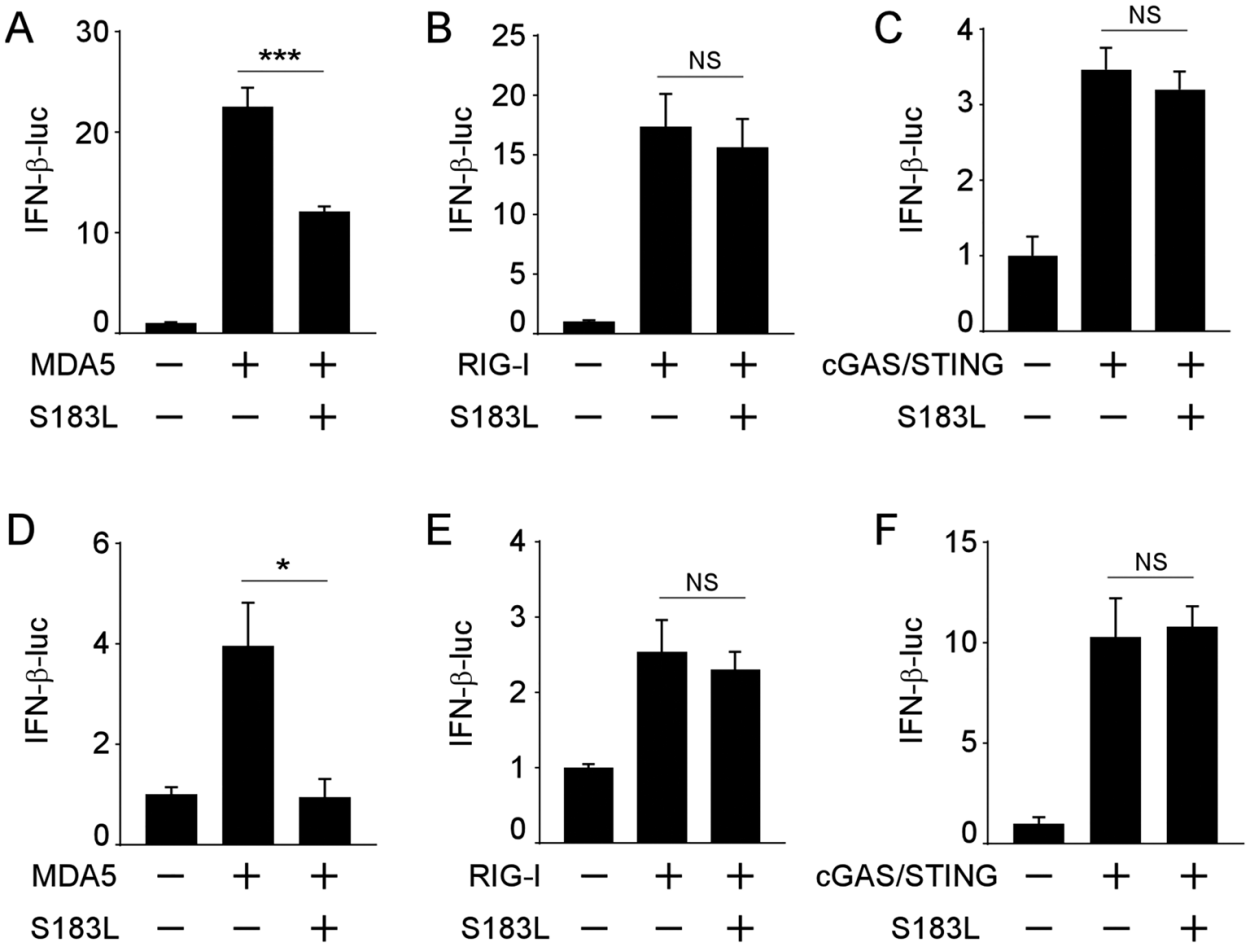
**pS183L inhibits MDA5-mediated IFN-β activation.** **A**–**C** 293 T cells were co-transfected with pGL3-Basic-IFN-β-Luc, pCMV-RL, pcDNA3-2XFlag-S183L or empty vector, along with pCAGGS-HA-MDA5 (**A**), pcDNA4-HA-RIG-I (**B**) or pcDNA4-HA-cGAS/pcDNA3-2XFlag-STING (**C**). 24 h post-transfection, the cells were collected for measuring luciferase activities. **D**–F The experiment was performed as shown in (**A**–**C**) except using PK15 cells.

Several ASFV-encoded proteins were screened using a dual-luciferase reporter assay to identify which ASFV proteins regulate the MDA5-mediated immune response. For this purpose, each viral protein was transfected with the IFN-β luciferase reporter along with an MDA5 stimulator. Among these, pS183L was identified (Additional file 1). To confirm the outcome, a dual-luciferase reporter assay was conducted by co-transfecting pGL3-Basic-IFN-β-Luc, pCMV-RL, along with MDA5/RIG-I/cGAS-STING and/or S183L expression vectors in 293 T and PK15 cells. The results showed that pS183L attenuated MDA5-mediated IFN-β promoter activity (Figures 1A and D) but not RIG-I (Figures 1B and E) or cGAS-STING-mediated IFN-β promoter activity (Figures 1C and F). Therefore, these results suggested that pS183L might inhibit the MDA5-mediated IFN-β signalling pathway.

### ASFV pS183L suppresses dsRNA-triggered IFN-β production

**Figure 2:**
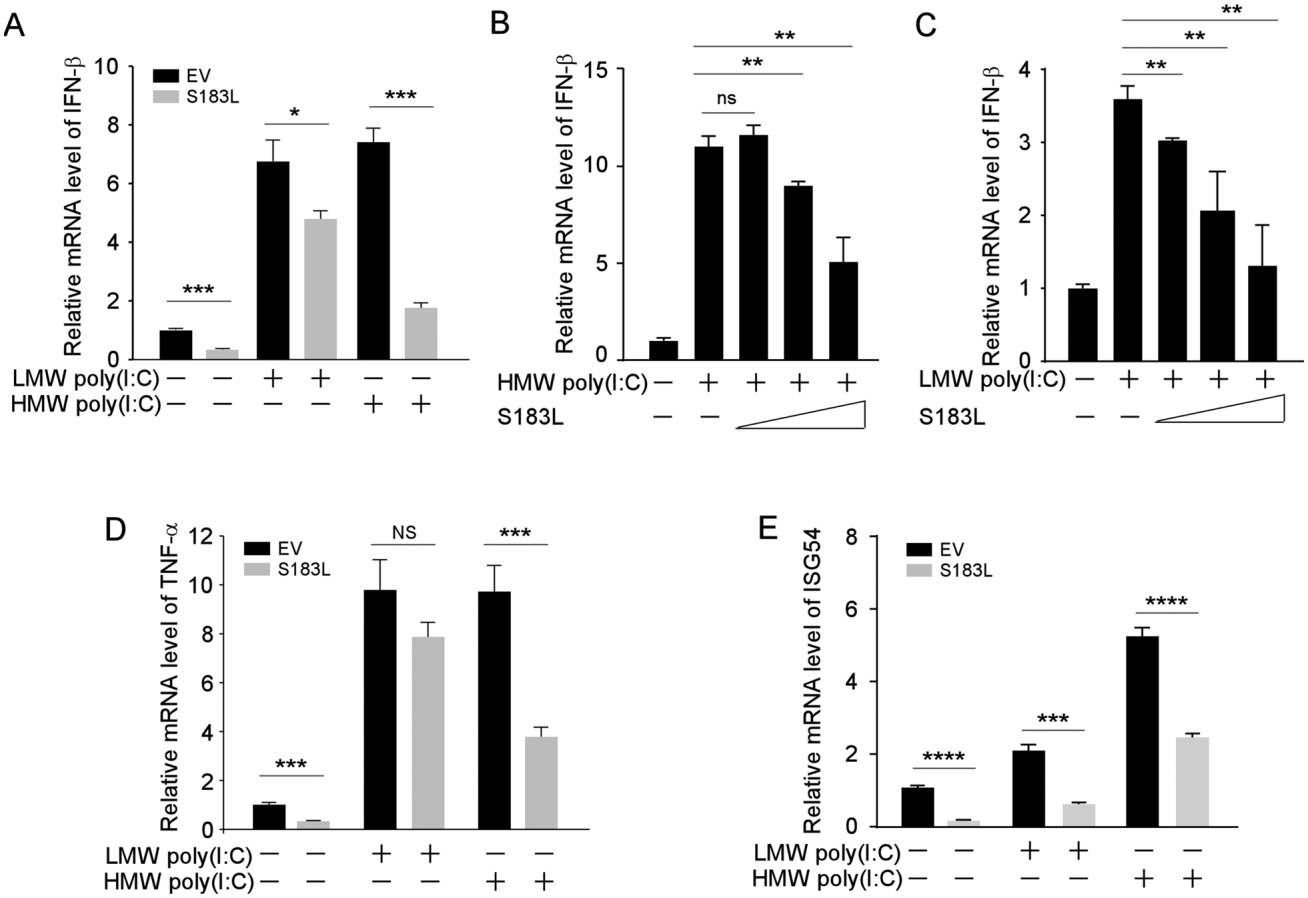
**pS183L suppresses IFN-β, TNF-α, and ISG54 mRNA expression level.** **A** PK15 cells were transfected with pcDNA3-2XFlag or pcDNA3-2XFlag-S183L for 24 h, followed by transfection of HMW poly(I:C) (10 μg/mL) or LMW poly(I:C) (10 μg/mL) for 1 h and then harvested for RNA extraction. Quantitative real-time PCR was carried out to detect IFN-β. (**B**-**C**) The experiment was performed as panel A, except that increasing amounts of pcDNA3-2XFlag-S183L were transfected. (**D**) The experiment was performed as panel A except that TNF-α mRNA expression level was detected. (**E**) PK15 cells were transfected with pcDNA3-2XFlag or pcDNA3-2XFlag-S183L for 24 h, followed by transfection of HMW poly(I:C) (10 μg/mL) or LMW poly(I:C) (10 μg/mL) for 4 h and then harvested for RNA extraction. Quantitative real-time PCR was carried out to detect ISG54.

While RIG-I recognises short synthetic dsRNA (< 0.5 kbp), MDA5 recognises long dsRNA (0.5–7 kbp) [36]. Thus, HMW poly(I:C) (1.5–8 kbp), synthetic analogues of dsRNA, will be recognised by MDA5 exclusively, while RIG-I predominantly recognises LMW poly(I:C) (0.2–1 kbp). To confirm the inhibitory effect of pS183L on MDA5-mediated IFN-β production, both LMW and HMW poly(I:C) were used to activate RIG-I or MDA5 after transfection with S183L or empty vector in PK15 cells. RT-qPCR was then used to check the transcriptional level of IFN-β. The results showed that overexpression of pS183L attenuated the levels of IFN-β mRNA with or without poly(I:C) treatment, particularly with the HMW poly(I:C) treatment (Figure 2A). The levels of IFN-β mRNA consistently declined as the expression of pS183L increased (Figures 2B and C). In response to cytosolic dsRNA, the downstream transcription factors, NF-κB and IRF3, induced the production of inflammatory cytokines and interferon-stimulated genes (ISGs). Therefore, the mRNA levels of inflammatory cytokine TNF-α and the ISG54 were determined when pS183L was overexpressed.

The results of this study demonstrate that both TNF-α and ISG54 mRNA levels decreased in the presence of pS183L (Figures 2D and E). Interestingly, pS183L was shown to exert a dual inhibitory effect on the signalling cascades activated by LMW poly(I:C) and HMW poly(I:C). Consequently, we investigated the RIG-I and MDA5 expression levels in response to LMW and HMW poly(I:C).

Our findings indicate that both LMW and HMW poly(I:C) induce an up-regulation of MDA5 mRNA and protein expression (Additional file 2). As reported in previous studies, this up-regulation is also observed in mesenchymal stem cells (MSCs) [37]. This outcome may also elucidate why pS183L specifically down-regulates the IFNβ luciferase reporter activity induced by MDA5 stimulation while inhibiting both LMW and HMW poly(I:C)-stimulated signalling. Furthermore, pS183L may also target RIG-I, potentially influencing its RNA recognition or other mechanisms that require further investigation. Together, these results suggest that pS183L regulates RLR signalling by targeting the MDA5-mediated pathway.

### ASFV pS183L inhibits the RLR signalling between MDA5 and MAVS

**Figure 3:**
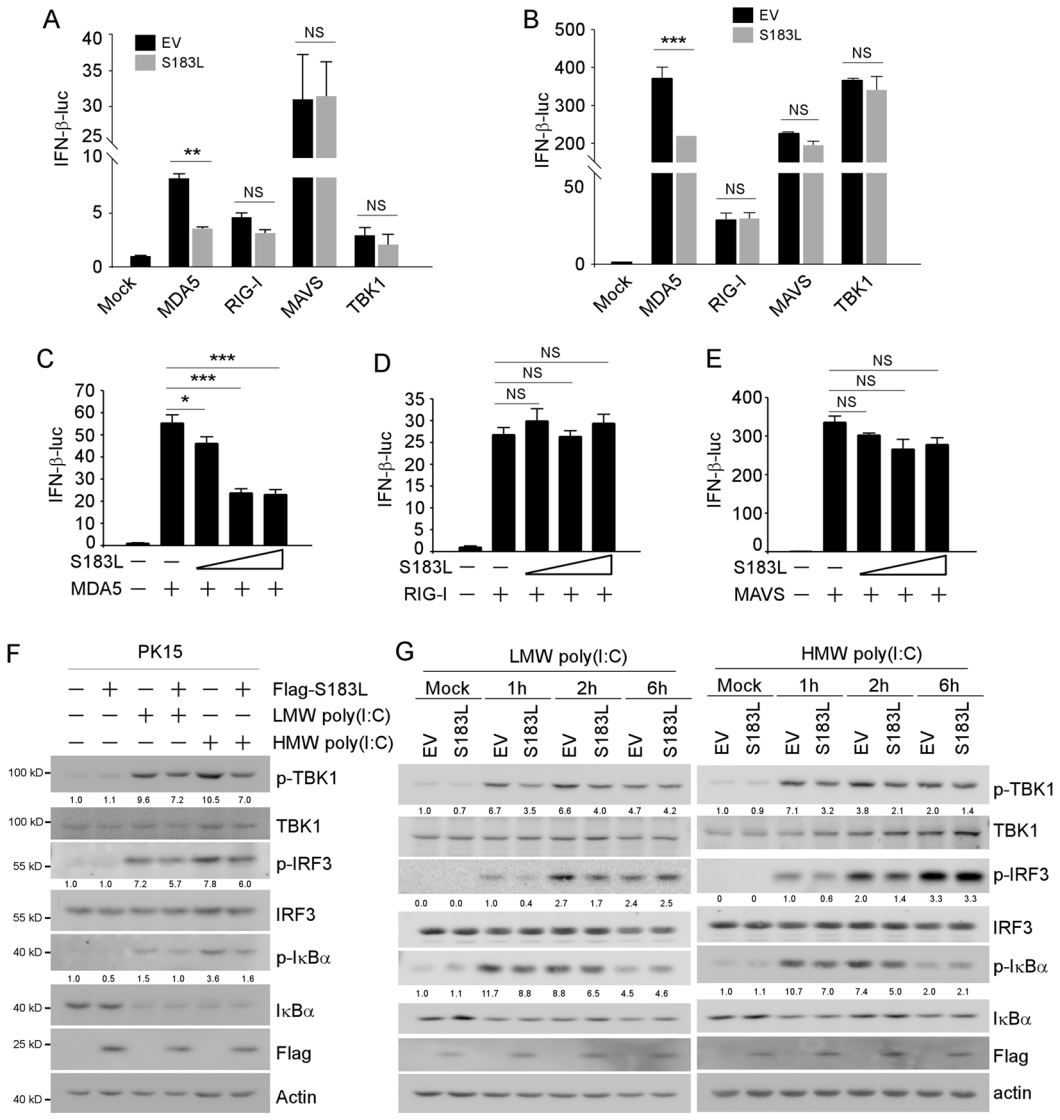
**pS183L suppresses IFN-β activation at or downstream of MDA5 and upstream of MAVS.** PK15 cells (**A**) or 293 T cells (**B**) were transfected with pGL3-Basic-IFN-β-Luc, pCMV-RL, pcDNA3-2XFlag-S183L or empty vector along with MDA5, RIG-I, MAVS or TBK1 expression vector. The cells were collected 24 h post-transfection, and luciferase activities were measured. (**C**-**E**) PK15 cells were transfected with pGL3-Basic-IFN-β-Luc, pCMV-RL, and an increasing dose of pcDNA3-2XFlag-S183L along with MDA5 (**C**), RIG-I (**D**) or MAVS (**E**) expression vector. Then, the cells were collected 24 h post-transfection and luciferase activities were measured. (**F**) PK15 cells were transfected with pcDNA3-2XFlag-S183L or empty vector for 24 h, followed by HMW poly(I:C) (10 μg/mL) or LMW poly(I:C) (10 μg/mL) transfection for 1 h. The cells were collected and analysed by immunoblotting with the indicated antibodies. (**G**) PK15 cells were transfected with pcDNA3-2XFlag-S183L or empty vector for 24 h, followed by LMW poly(I:C) (10 μg/mL) or HMW poly(I:C) (10 μg/mL) transfection for 1, 2 and 6 h. The cells were collected and analysed by immunoblotting with the indicated antibodies. The levels of phosphorylated proteins were normalised to the total protein levels.

The association of viral dsRNA with RLRs induces RIG-I or MDA5 conformational changes and oligomerisation, activating the mitochondrial protein MAVS and cytosolic kinase TBK1 to stimulate IFN-β signalling. This study used different factors along the signalling cascade to activate the RLRs signalling to characterise the specific target of pS183L, RIG-I, MDA5, MAVS, and TBK1. The dual luciferase reporter assay results indicated that pS183L attenuated the activation of the IFN-β promoter by MDA5 but not by RIG-I, MAVS, or TBK1). This finding was observed in both PK15 (Figure 3A) and 293 T cells (Figure 3B). Consequently, various doses of S183L plasmids were transfected into PK15 cells following stimulation with RIG-I, MDA5, or MAVS to confirm this result further.

The study’s findings showed that pS183L consistently inhibited MDA5-mediated activation (Figure 3C) but not RIG-I- (Figure 3D) or MAVS- (Figure 3E) mediated activation of the IFN-β promoter in a dose-dependent manner. These results demonstrate that pS183L inhibits IFN-β-targeting at, or downstream, of MDA5 and upstream of MAVS. Immunoblotting results showed that poly(I:C) stimulated p-TBK1, p-IRF3 and p-IκBα was attenuated upon pS183L overexpression in PK15 cells, especially with HMW poly(I:C) treatment (Figure 3F). Various durations of stimulation with LMW poly(I:C) or HMW poly(I:C) demonstrated that pS183L exerts a time-dependent inhibitory effect on the RLR signalling pathway (Figure 3G). Generally, the down-regulation of p-TBK1 following pS183L overexpression suggests that the target of pS183L is situated upstream of TBK1, which aligns with the dual-luciferase results (Figures 3A–E).

Collectively, the results show that pS183L inhibits MDA5-mediated IFN-β-signalling at or downstream of MDA5 and upstream of MAVS.

### ASFV pS183L interacts with MDA5

The RNA sensor MDA5 recruits MAVS to initiate type I IFN signalling upon detecting viral RNAs. Considering that pS183L inhibits IFN-β production at or downstream of MDA5 and upstream of MAVS, we hypothesise that pS183L targets MDA5. Consequently, a co-immunoprecipitation assay was conducted to analyse the protein–protein interaction between pS183L and MDA5 by co-transfecting plasmids that express MDA5 and S183L in 293 T cells.

**Figure 4:**
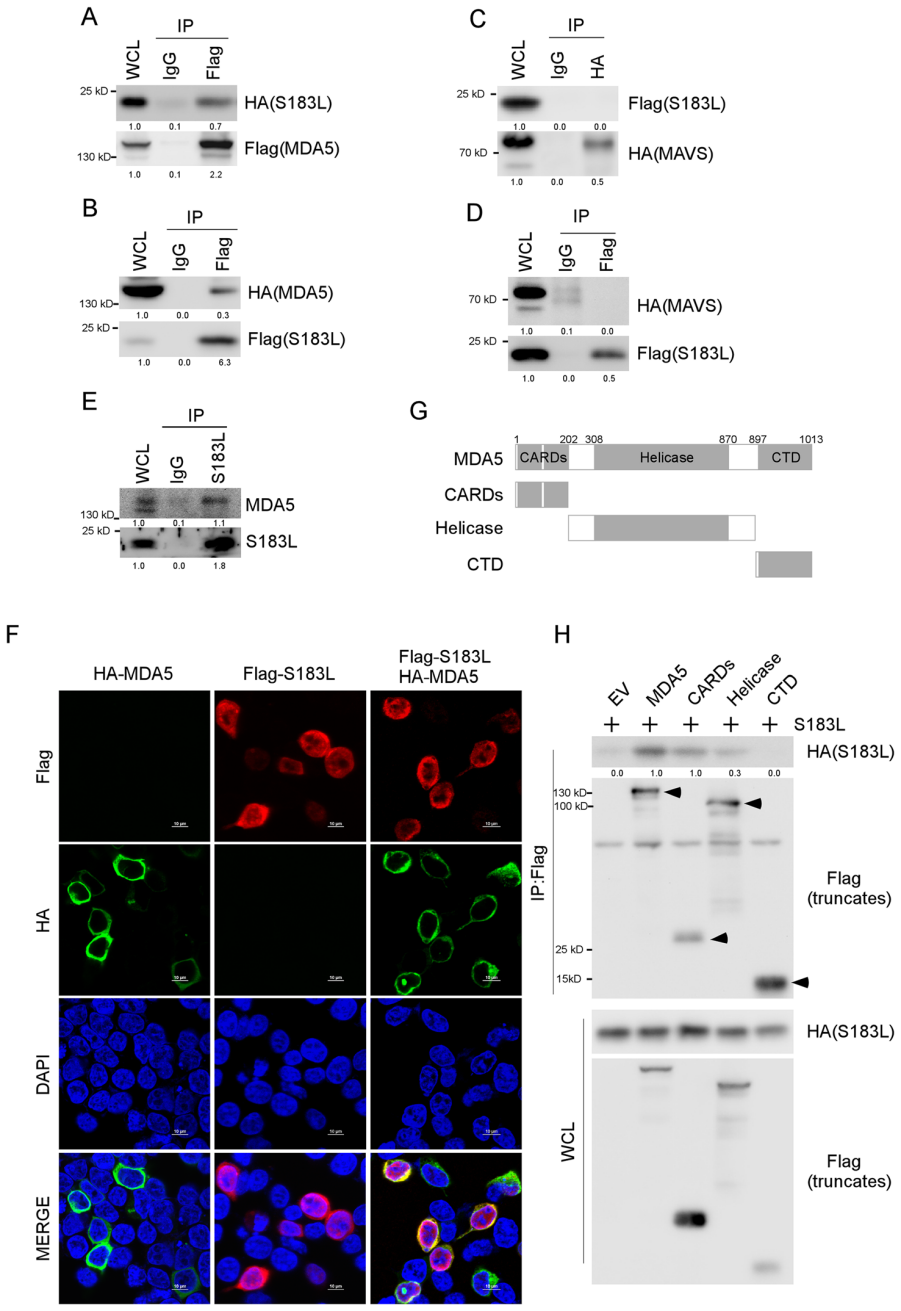
**pS183L interacts with MDA5 CARD and Helicase domains.** **A** A co-immunoprecipitation assay was performed with whole cell lysates prepared with 293 T cells co-transfected with Flag-MDA5 and HA-S183L for 24 h with Flag antibodies or control IgG. The immunocomplexes were analysed by immunoblotting with the indicated antibodies. **B** The experiment was performed as for panel A, except that Flag-S183L and HA-MDA5 were transfected. **C**–**D** The experiment was performed as for panel A or B, except that HA-MAVS and Flag-S183L were transfected. **E** A co-immunoprecipitation assay was performed with whole cell lysates prepared with PAMs infected with ASFV (MOI, 1) for 24 h with pS183L antibody. The immunocomplexes were analysed by immunoblotting with the indicated antibodies. **F** 293 T cells were transfected with Flag-S183L and HA-MDA5 for 24 h and stained with anti-Flag (red) and anti-HA (green) antibodies. The nuclei were stained with DAPI (blue), and images were acquired with confocal microscopy. **G** Diagrams of MDA5 truncates. **H** A co-immunoprecipitation assay was performed with whole cell lysates prepared with 293 T cells co-transfected with HA-S183L and Flag-MDA5 for 24 h with Flag antibodies. The immunocomplexes were analysed by immunoblotting with the indicated antibodies. The levels of the interacting proteins were normalised to the levels of the whole cell lysate proteins (**A**–**E**) or proteins immunoprecipitated by the antibody (**H**).

Our results revealed the presence of HA-tagged S183L in the Flag antibodies immunoprecipitated MDA5 protein complex (Figure 4A). Conversely, HA-tagged MDA5 was detected in the Flag antibodies immunoprecipitated pS183L protein complex (Figure 4B). However, pS183L was not detected in the MAVS immunoprecipitated complex (Figure 4C), and MAVS was not present in the pS183L immunoprecipitated complex (Figure 4D). A co-immunoprecipitation assay was subsequently performed to verify the interactions between pS183L and MDA5 in ASFV-infected PAM cells. The outcome of the co-immunoprecipitation assay indicated that endogenous MDA5 was present in the pS183L antibodies immunoprecipitated complex (Figure 4E).

Furthermore, an indirect immunofluorescence assay showed that pS183L localised both in the cytoplasm and nucleus in 293 T cells, while pS183L co-localised with MDA5 in the cytoplasm (Figure 4F). As pS183L is an uncharacterised protein, it may play a role in the nucleus during ASFV infection, except immune evasion. MDA5 comprises two caspase activation and recruitment domains (CARD), a central helicase domain and a CTD. The CARD domain of MDA5 is reported to interact with MAVS and activate the downstream signalling [38]. The helicase domain is responsible for RNA-dependent ATP hydrolysis, and the CTD domain is capable of binding to dsRNA [30, 39].

Flag-tagged MDA5 truncations containing CARD, helicase, or CTD domains were constructed (Figure 4G), and a co-immunoprecipitation assay was performed to analyse which domain of MDA5 interacts with pS183L. The co-immunoprecipitation results indicated that HA-tagged S183L were identified in Flag antibodies that precipitated the CARD and helicase domains of MDA5 (Figure 4H). Previous reports have demonstrated that the CARD domain of MDA5 is phosphorylated in uninfected cells and dephosphorylated by PP1α and PP1γ upon virus infection or dsRNA stimulation, which leads to a structural change within the tandem CARDs [40].

Notably, the CARD domain of MDA5 binds to K63-linked polyubiquitin chains to activate the immune response [39–42]. Furthermore, it was reported that the CARDs of MDA5 interact with the CARD of MAVS [43, 44], which suggests that pS183L may target the CARD domain of MDA5 to disrupt the interaction between MDA5 and MAVS, further inhibiting IFN-β production.

### pS183L inhibits MDA5 oligomerisation

**Figure 5:**
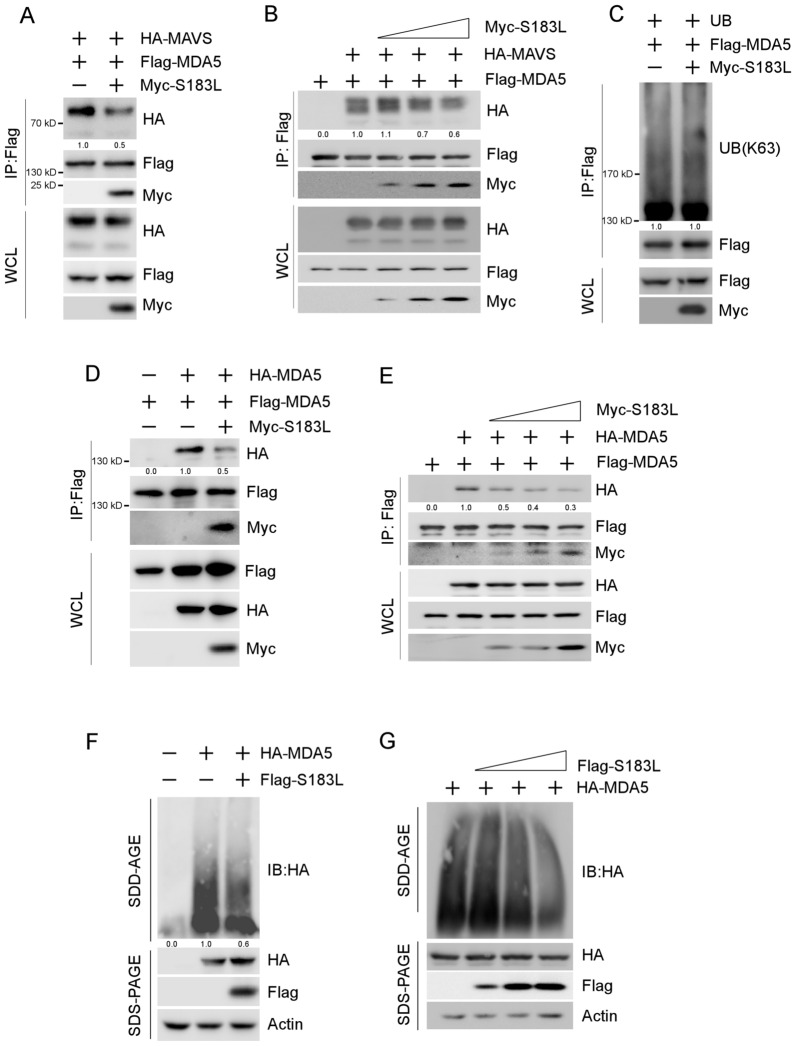
**pS183L blocks MDA5 oligomerisation.** **A** Co-immunoprecipitation assay was performed with whole cell lysates prepared with 293 T cells co-transfected with pcDNA4-HA-MAVS, pcDNA3-2XFlag-MDA5 and pcDNA3-Myc-S183L or empty vector for 24 h with Flag antibodies. The immunocomplexes were analysed by immunoblotting with the indicated antibodies. **B** The experiment was performed as panel A, except that increasing amounts of pcDNA3-2XFlag-S183L were transfected. **C** Immunoprecipitation assay was performed with whole cell lysates prepared with 293 T cells co-transfected with pcDNA3-UB, pcDNA3-2XFlag-MDA5 and pcDNA4-HA-S183L or empty vector for 24 h with Flag antibodies. The immunocomplexes were analysed by immunoblotting with the indicated antibodies. **D** Co-immunoprecipitation assay was performed with whole cell lysates prepared with 293 T cells co-transfected with pcDNA4-HA-MDA5, pcDNA3-2XFlag-MDA5 and pcDNA3-Myc-S183L or empty vector for 24 h with Flag antibodies. The immunocomplexes were analysed by immunoblotting with the indicated antibodies. **E** The experiment was performed as panel D, except that increasing amounts of pcDNA3-2XFlag-S183L were transfected. **F** 293 T cells were co-transfected with pcDNA4-HA-MDA5 and pcDNA3-2XFlag-S183L or empty vector for 24 h. Whole-cell extracts were prepared and analysed by immunoblotting and SDD-AGE. **G** The experiment was performed as panel F, except that increasing amounts of pcDNA3-2XFlag-S183L were transfected. The interacting proteins’ levels were normalised to those of the proteins immunoprecipitated by the respective antibodies

A co-immunoprecipitation assay was conducted to investigate the role of pS183L in the interaction between MDA5 and MAVS. The results indicated that HA-tagged MAVS decreased upon S183L overexpression in the precipitated Flag-tagged MDA5 protein complex (Figure 5A). In addition, the increasing dose of pS183L gradually decreased HA-tagged MAVS (Figure 5B). Once MDA5 is activated by viral RNA in the cytoplasm, it binds with unanchored lysine-63 (K63) polyubiquitin chains and initiates MDA5 oligomerisation to recruit the signalling adaptor MAVS [39, 41, 42, 44, 45]. Therefore, exploring how pS183L regulates MDA5 to interfere with the recruitment of MAVS is valuable.

The K63 polyubiquitination of MDA5 was determined by co-transfecting Flag-tagged MDA5 and ubiquitin (UB) with or without pS183L expression in 293 T cells. Our findings revealed that pS183L expression did not affect the K63 polyubiquitination level of MDA5 (Figure 5C). To further assess whether pS183L regulates the oligomerisation of MDA5, a co-immunoprecipitation assay and semi-denaturing detergent agarose gel electrophoresis (SDD-AGE) assay were conducted. The results showed that pS183L disrupted the interaction between Flag-tagged MDA5 and HA-tagged MDA5 (Figure 5D) as well as MDA5 oligomerisation levels (Figure 5F). In addition, the interaction between Flag-tagged MDA5 and HA-tagged MDA5 and MDA5 oligomerisation levels was gradually inhibited with the increasing dose of pS183L (Figures 5E–G).

These data suggest that ASFV pS183L attenuates IFN-β production via interacting with MDA5 and subsequently blocking MDA5 oligomerisation and recruitment of MAVS.

## Discussion

Although ASF was reported a century ago, no safe and effective vaccine is available. ASFV has a large genome and complex structure. It encodes over 150 proteins, most of which regulate the host’s immune response through different mechanisms, such as apoptosis, autophagy, the inflammatory response, and type I interferon regulation [9]. However, the function of many viral immune regulators remains unknown. This study found that pS183L, an uncharacterised ASFV viral protein, negatively regulates innate immune response by targeting MDA5. Importantly, to our knowledge, this is the first evidence that ASFV protein regulates MDA5-mediated IFN-β production.

As a DNA virus, the interaction between ASFV and the cytosolic DNA-sensing cGAS-STING pathway has been extensively studied. Findings from these studies indicate that at least 20 ASFV-encoded proteins suppress the cGAS-STING pathway by regulating the oligomerisation, phosphorylation, ubiquitination, or degradation of key signal transducers, including STING, TBK1, IRF3 and others [12, 46, 47]. Interestingly, the mRNA levels of RNA sensors RIG-I and MDA5 were found to increase upon ASFV infection at early time points but decrease along with the progression of viral replication [28, 35]. This outcome indicates that RNA intermediates produced during ASFV may interact with the RIG-I and MDA5-mediated immune response. Indeed, it has been shown that ASFV I267L suppresses RNA polymerase III-RIG-I-mediated innate immunity [16].

Nevertheless, there is no report that ASFV-encoded proteins regulate the MDA5-mediated immune signalling. However, it has been demonstrated that the dsDNA virus HSV-1 US11 binds to MDA5 to disrupt the MDA5-MAVS complex and suppress the production of type I IFN [48]. We found that pS183L not only inhibits MDA5-mediated activation of the IFN-β promoter but also down-regulates poly(I:C)-induced IFN-β transcription in PK15 cells (Figures 1D and 2A). Therefore, we hypothesised that pS183L is a potential inhibitor of RLR-mediated antiviral signalling.

MDA5 is a critical cytoplasmic dsRNA receptor essential for transducing danger signalling to initiate IFN-β production. In this study, we showed that pS183L interacts with the CARD domain of MDA5 (Figures 4A, B, and G) but not with that of MAVS (Figures 4C and D). This finding shows a 19% similarity in the CARD domain with MDA5. Our results also showed that pS183L interacted with RIG-I (Additional file 3), suggesting an unknown interaction mechanism may exist between pS183L and RIG-I.

Once it recognises viral RNAs, MDA5 undergoes a conformational change that exposes and multimers its CARD domains, allowing for CARD-CARD interactions with MAVS [49]. Consequently, the role of pS183L in MDA5 oligomerisation was explored. The results indicated that pS183L-MDA5 interaction suppresses the MDA5 oligomerisation, further blocking the interaction between MDA5 and MAVS (Figure 5). Interestingly, in addition to binding with the CARD domain of MDA5, pS183L also interacts with its helicase domain. Generally, MDA5 senses viral RNA through its helicase domain and transmits a signal downstream via the CARD domain. Therefore, the interaction between pS183L and the MDA5’s helicase domain could potentially disrupt the viral RNA sensing mechanism and further block the MDA5 signalling activation.

Studies have indicated that RIG-I and MDA5 are major sensors of viral RNAs derived from viruses, especially RNA viruses such as Influenza A, Dengue, and Zika [50–53]. Interestingly, host-derived RNAs also activate the RLRs signalling upon virus infection. For example, HSV-1 infection results in mis-localisation and unmasking of cellular non-coding RNAs, which interact and activate RIG-I [25]. Similarly, host RNAs are recognised by RLRs to activate the immune response upon KSHV infection [27]. Both RIG-I and MDA5 are activated by ASFV infection, but the mechanism remains unknown [28, 35]. Recently, OAS1(2′, 5′-oligoadenylate synthetase gene 1), an interferon-stimulating gene effector, was reported to be up-regulated 24 h post-ASFV infection [54]. OAS1 was also found to regulate the cleavage of cellular RNA by RNase L to activate the RIG-I and MDA5 signalling pathway [55]. However, it remains unknown how RIG-I and MDA5 are stimulated at the early stage of ASFV infection and down-regulated at the later stage. Therefore, other host or viral factors involved in the signalling of RIG-I or MDA5 upon ASFV infection deserve further exploration.

Vaccines are the most effective way to prevent and control ASFV infections, but the development of vaccines is hindered by the lack of understanding of many virulence genes, essential viral replication genes, and key genes that regulate the host immune response. In our previous studies, we found that pI73R and pCP312R are determined to be Z-DNA binding or ssDNA binding proteins [56, 57]. Furthermore, it has been reported that I73R is a virulence-related gene, and the deletion of I73R provides complete homologous protection for pigs [58]. Importantly, we also found that viral exonuclease pD345L, encoded by one replication-essential gene, can inhibit NF-κB signalling by blocking IKK kinase activity [32].

Moreover, some replication-essential genes, such as E301R and S273R, were reported to suppress IFN-β by targeting IRF3 or the IKK complex [59, 60]. These findings could partially explain why the live attenuated ASFV strains still maintain immune suppression activities. Given that pS183L is the first ASFV protein to target MDA5 for down-regulating RLR signalling, our subsequent studies will focus on generating the S183L deletion virus to evaluate its role in viral replication in vitro and in vivo.

## Supplementary Information

**Additional file 1. Screening of ASFV opening reading frames (ORFs) that modulate MDA5-mediated transactivation of the IFN-β promoter. **293T cells were co-transfected with pGL3-Basic-IFN-β-Luc, pCMV-RL, Flag-tagged ASFV ORFs or empty vector, and pCAGGS-HA-MDA5. 24 h post-transfection, the cells were collected to measure luciferase activities.

**Additional file 2. Both LMW poly(I:C) and HMW poly(I:C) up-regulate MDA5 and RIG-I transcription. **PK15 cells were transfected with LMW poly(I:C) or HMW poly(I:C) (10 μg/mL) for the indicated times. The cells were then harvested for RNA extraction. Semiquantitative PCR was carried out to detect MDA5 and RIG-I.

**Additional file 3. pS183L interacts with RIG-I. **(A) A co-immunoprecipitation assay was performed with whole cell lysates prepared with 293T cells co-transfected with Flag-RIG-I and HA-S183L for 24 h with Flag antibodies or control IgG. The immunocomplexes were analysed by immunoblotting with the indicated antibodies. (B) The experiment was performed as for panel A, except that Flag-S183L and HA-RIG-I were transfected.

## Data Availability

All data generated or analysed during this study are included in this published article.
