# Supplementary material for: ASFV pS183L protein negatively regulates RLR-mediated antiviral signalling by blocking MDA5 oligomerisation
Source: Vet Res. 2025 Mar 31;56:70. doi: 10.1186/s13567-025-01488-x (PMC11959855; doi:10.1186/s13567-025-01488-x)
Supplement: Supplementary file 1 — Additional file 1. Screening of ASFV opening reading frames (ORFs) that modulate MDA5-mediated transactivation of the IFN-β promoter. 293T cells were co-transfected with pGL3-Basic-IFN-β-Luc, pCMV-RL, Flag-tagged ASFV ORFs or empty vector, and pCAGGS-HA-MDA5. 24 h post-transfection, the cells were collected to measure luciferase activities. [file 13567_2025_1488_MOESM1_ESM.doc]

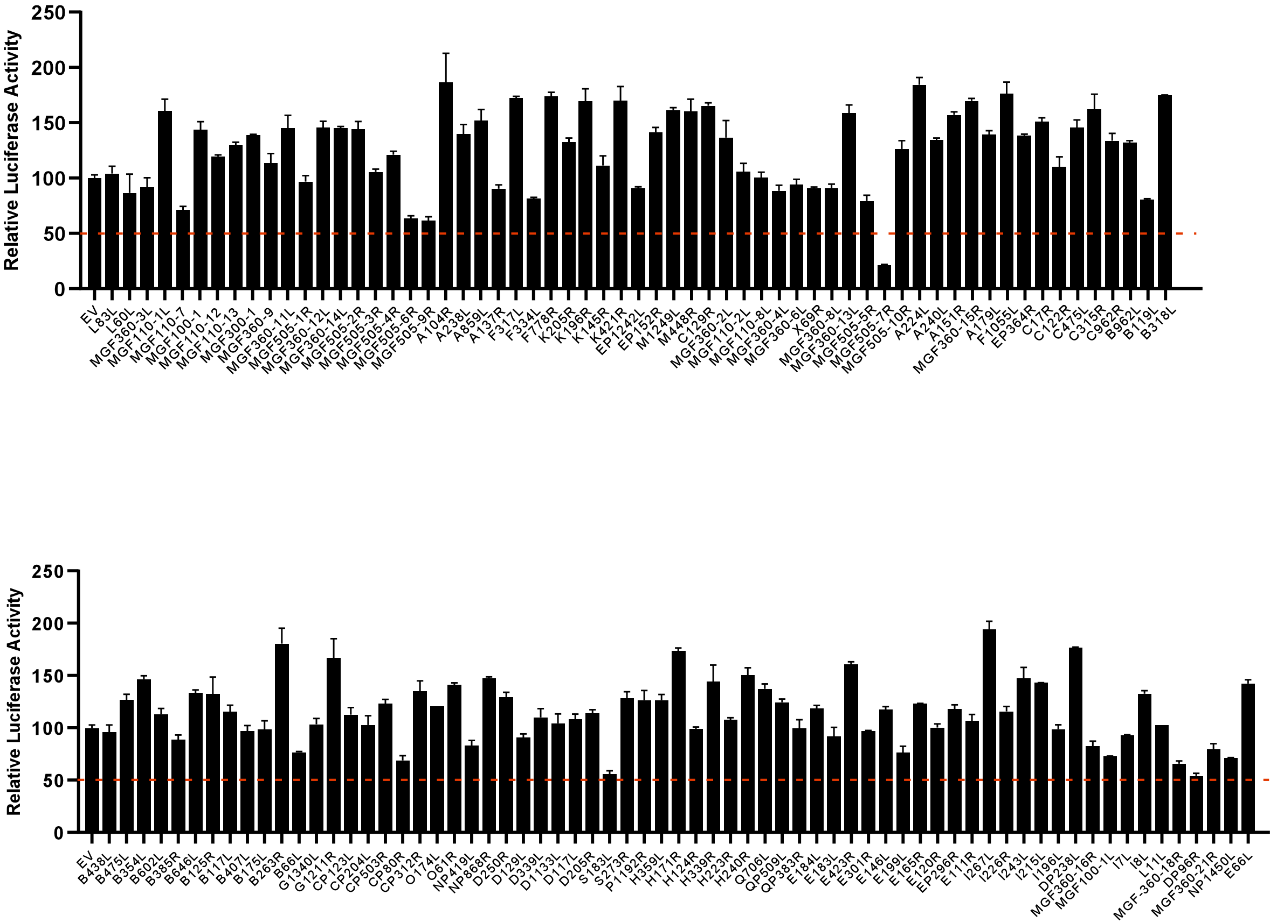


**Additional file 1. Screening of ASFV opening reading frames (ORFs) that modulate MDA5-mediated transactivation of the IFN-β promoter.** 293T cells were co-transfected with pGL3-Basic-IFN-β-Luc, pCMV-RL, Flag-tagged ASFV ORFs or empty vector, along with pCAGGS-HA-MDA5. 24 h post transfection, the cells were collected for measuring luciferase activities.
