# Supplementary material for: ASFV pS183L protein negatively regulates RLR-mediated antiviral signalling by blocking MDA5 oligomerisation
Source: Vet Res. 2025 Mar 31;56:70. doi: 10.1186/s13567-025-01488-x (PMC11959855; doi:10.1186/s13567-025-01488-x)
Supplement: Supplementary file 2 — Additional file 2. Both LMW poly(I:C) and HMW poly(I:C) up-regulate MDA5 and RIG-I transcription. PK15 cells were transfected with LMW poly(I:C) or HMW poly(I:C) (10 μg/mL) for the indicated times. The cells were then harvested for RNA extraction. Semiquantitative PCR was carried out to detect MDA5 and RIG-I. [file 13567_2025_1488_MOESM2_ESM.doc]

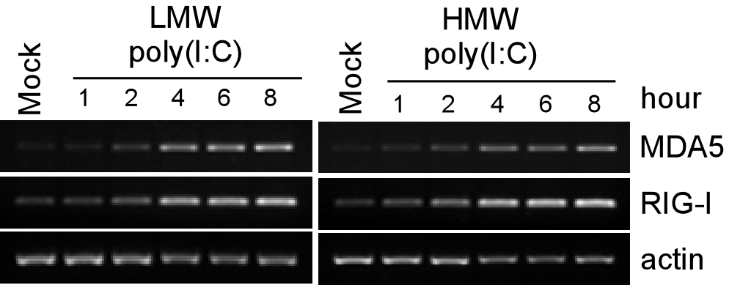


**Additional file 2. Both LMW poly(I:C) and HMW poly(I:C) up-regulate MDA5 and RIG-I transcription.** PK15 cells were transfected with LMW poly(I:C) or HMW poly(I:C) (10 μg/mL) for the indicated times. The cells were then harvested for RNA extraction. Semiquantitative PCR was carried out to detect MDA5 and RIG-I.
