# Supplementary material for: ASFV pS183L protein negatively regulates RLR-mediated antiviral signalling by blocking MDA5 oligomerisation
Source: Vet Res. 2025 Mar 31;56:70. doi: 10.1186/s13567-025-01488-x (PMC11959855; doi:10.1186/s13567-025-01488-x)
Supplement: Supplementary file 3 — Additional file 3. pS183L interacts with RIG-I. (A) A co-immunoprecipitation assay was performed with whole cell lysates prepared with 293T cells co-transfected with Flag-RIG-I and HA-S183L for 24 h with Flag antibodies or control IgG. The immunocomplexes were analysed by immunoblotting with the indicated antibodies. (B) The experiment was performed as for panel A, except that Flag-S183L and HA-RIG-I were transfected. [file 13567_2025_1488_MOESM3_ESM.doc]

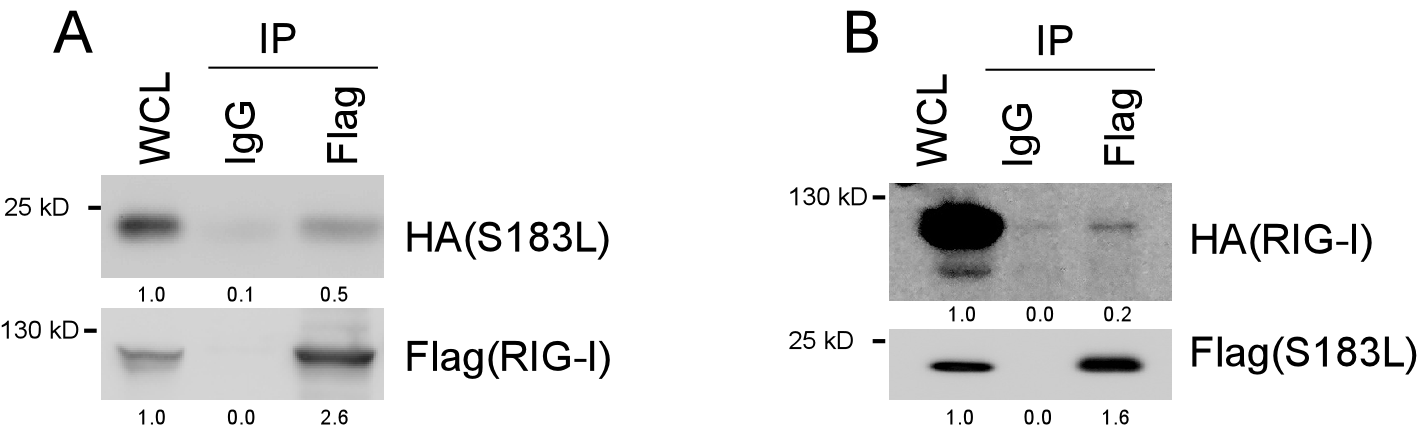


**Additional file 3. pS183L interacts with RIG-I.** (A) Co-immunoprecipitation assay was performed with whole cell lysates prepared with 293T cells co-transfected with Flag-RIG-I and HA-S183L for 24 h with Flag antibodies or control IgG. The immunocomplexes were analyzed by immunoblotting with the indicated antibodies. (B) The experiment was performed as for panel A, except that Flag-S183L and HA-RIG-I were transfected.
